# Proteostasis Dysfunction in Aged Mammalian Cells. The Stressful Role of Inflammation

**DOI:** 10.3389/fmolb.2021.658742

**Published:** 2021-06-17

**Authors:** Diego Ruano

**Affiliations:** ^1^ Instituto de Biomedicina de Sevilla (IBiS), Hospital Universitario Virgen del Rocío/Consejo Superior de Investigaciones Científicas/Universidad de Sevilla, Sevilla, Spain; ^2^ Departamento de Bioquímica y Biología Molecular, Facultad de Farmacia, Universidad de Sevilla, Sevilla, Spain

**Keywords:** aging, proteasome, autophagy, inflammation, proteostasis, cell stress and aging

## Abstract

Aging is a biological and multifactorial process characterized by a progressive and irreversible deterioration of the physiological functions leading to a progressive increase in morbidity. In the next decades, the world population is expected to reach ten billion, and globally, elderly people over 80 are projected to triple in 2050. Consequently, it is also expected an increase in the incidence of age-related pathologies such as cancer, diabetes, or neurodegenerative disorders. Disturbance of cellular protein homeostasis (proteostasis) is a hallmark of normal aging that increases cell vulnerability and might be involved in the etiology of several age-related diseases. This review will focus on the molecular alterations occurring during normal aging in the most relevant protein quality control systems such as molecular chaperones, the UPS, and the ALS. Also, alterations in their functional cooperation will be analyzed. Finally, the role of inflammation, as a synergistic negative factor of the protein quality control systems during normal aging, will also be addressed. A better comprehension of the age-dependent modifications affecting the cellular proteostasis, as well as the knowledge of the mechanisms underlying these alterations, might be very helpful to identify relevant risk factors that could be responsible for or contribute to cell deterioration, a fundamental question still pending in biomedicine.

## Introduction

### Protein Quality Control Systems

Proteostasis is the dynamic regulation of a balanced, functional proteome, in order to maintain its functionality. In eukaryotic cells, proteostasis is maintained by different quality control systems such as molecular chaperones, the UPS, and the ALS. The correct function and coordination of all of them guarantee that proteins can be properly synthesized, folded, assembled, sub-compartmentalized, and finally degraded according to cellular requirements.

### Molecular Chaperones

Molecular chaperones are ubiquitous and highly conserved proteins. They include an array of different molecular weight proteins, ranging from ten to more than 100 kDa, distributed in different cellular compartments (Kampinga et al., 2009; Ciechanover and Kwon 2017). In particular, the human chaperome involves 332 genes that can be grouped into nine functional families: HSP90, HSP70, HSP60, HSP40, small HSPs, tetratricopeptide repeat-domain-containing, prefoldin, and ER and mitochondria specific chaperones (see Brehme et al., 2014 for a detailed review). Molecular chaperones promote efficient *de novo* protein folding, prevent aggregation of mis/unfolded proteins (Hartl et al., 2011; Kim Y.,E. et al., 2013), disaggregate aggregated proteins (Weibezahn et al., 2005), and target misfolded proteins for refolding or protein degradation (Pickart and Cohen, 2004). They bind to substrate proteins through exposed hydrophobic regions and/or unstructured polypeptide backbones, two hallmarks of non-native conformations. For example, HSP70, one of the most abundant cellular chaperones, participates in *de novo* protein folding, post-translational refolding of aggregation-prone proteins, and the re-solubilization of protein aggregates (Mayer and Gierasch, 2019). In summary, chaperones have pivotal roles in proteostasis from protein synthesis to protein degradation.

### The Ubiquitin Proteasome System

The UPS is responsible for the catalysis of the ATP-dependent degradation of most of the soluble and short-lived poly-ubiquitinated proteins by the 20S proteasome. The 20S proteasome is a hollow barrel-shaped structure built of four rings: two outer α-rings, and two inner β-rings, each one containing seven subunits (αl to α7 or β1 to β7). The α-rings control the substrate access to the proteolytic chamber, whereas the β-rings harbor the constitutive catalytic subunits: β_1_ (caspase-like activity), β_2_ (trypsin-like activity), and β_5_ (chymotrypsin-like activity). The 20S proteasome mainly degrades either non-ubiquitinated misfolded, oxidized or damaged proteins (Raynes et al., 2016). However, the 20S proteasome (the proteolytic module) can associate with one or two terminal regulatory particle/s called 19S, giving rise to the 26S or 30S proteasome, respectively, which is responsible for the degradation of soluble and short-lived poly-ubiquitinated proteins (Rechsteiner and Hill, 2005; Ding and Yin, 2008; Jung and Grune, 2012). The 19S regulatory particle is terminally attached to the 20S core and is built of two different structures: a ring-shaped base and a mobile lid structure. The ring-shaped base is made up of ten different subunits: the Rpt1-Rpt6 ring, and Rpn1, Rpn2, Rpn10, and Rpn13), whereas the lid structure includes nine additional subunits (Rpn3, Rpn5, Rpn6, Rpn7, Rpn8, Rpn9, Rpn11, Rpn12, and Rpn15), each one with different functions (Tanaka 2009). For example, the Rpt1-Rpt6 ring has ATPase activity and regulates substrate unfolding and substrate transfer through the channel. (Collins and Goldberg, 2017). The Rpn10 and Rpn13 possess ubiquitin-binding domains and functions as receptor for ubiquitinated substrates, whereas the Rpn11 subunit is a de-ubiquitinating enzyme that removes poly-ubiquitin chains from target proteins, allowing the release and re-use of ubiquitin molecules (Finley, 2009). Thus, the 19S particle can bind poly-ubiquitinated proteins, catalyze protein de-ubiquitination, unfold the target protein, and promote protein degradation into the catalytic chamber.

**FIGURE 1:**
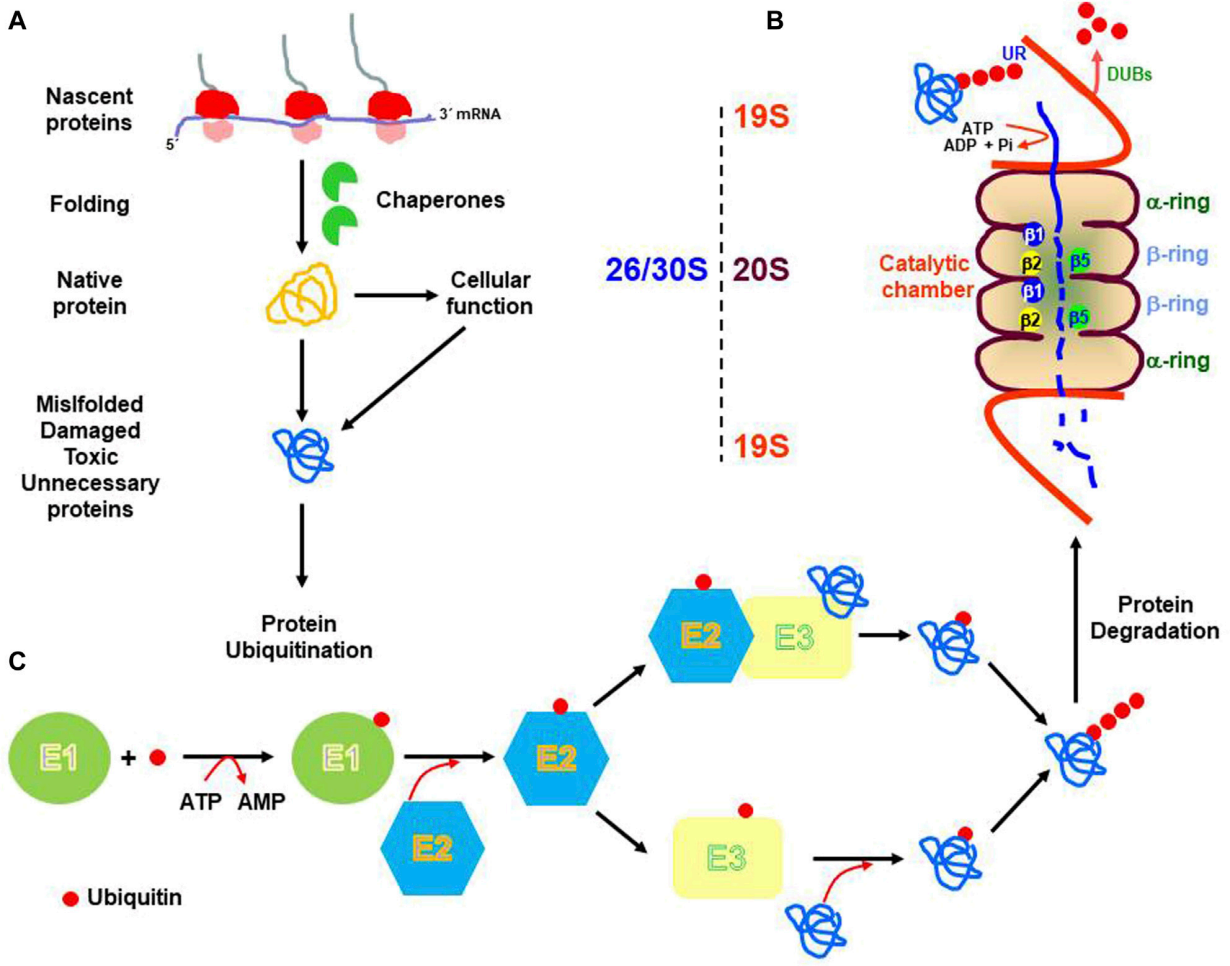
Schematic representation of the cellular biology of proteins. mRNA translation produces nascent proteins that are assisted during folding by chaperones. Native proteins perform their cellular functions and are subject to cellular turnover **(A)**. Also, unfolded, misfolded, oxidative damage, modified, or unnecessary proteins are targeted for degradation by the 26S or 30S proteasome [19S-20S or 19S-20S-19S, respectively: **(B)**]. Previous to proteasome degradation proteins need to be ubiquitinated by three enzymatic reactions catalyzed by the E1 (ubiquitin-activating), E2 ubiquitin-conjugating) and E3 (ubiquitin-ligase), respectively **(C)**. UR. Ubiquitin receptor; DUBs. Deubiquitinating enzymes. β1, β2, and β5. Catalytic subunits.

Proteins degraded by the 26S proteasome, need to be previously tagged with ubiquitin in a process called protein ubiquitination (Ciechanover and Kwon 2017). This process involves the binding of ubiquitin to the target protein. Ubiquitin is a small and conserved protein of 76-amino acid residue, with seven residues of lysine located at positions 6, 11, 27, 29, 33, 48, and 63. (Komander, 2009), that is mainly bound to lysine residues on the target protein, although it can also be attached to other residues such as Ser/Thr (Shimizu et al., 2010) Cys (Cadwell and Coscoy, 2005), or even to the N-terminus of the target protein (Breitschopf et al., 1998). First, ubiquitin is activated by the E1 enzyme (ubiquitin-activating), which catalyzes the ATP-dependent formation of a thioester bond between the C-terminal Gly carboxyl group of ubiquitin, and the Cys residue of the active site of the E1 enzyme. Then, ubiquitin is transferred to the Cys residue of a member of the E2 family of enzymes (ubiquitin-conjugating), and finally, substrate specificity is provided by specific E3 enzymes (ubiquitin ligase) of the E3 RING or HECT families. In general, four residues of ubiquitin bound through Lys-48 constitute the stronger degradation signal (Korovila et al., 2017) (see Figure 1 for a global overview).

In addition to the degradation of soluble cytosolic proteins, the UPS is also involved in the degradation of unfolded or misfolded proteins synthesized into the ER. It is estimated that around one-third of the total cellular proteins, secreted and transmembrane proteins, are synthesized and folded inside the ER (Braakman and Hebert, 2013). These proteins are especially dependent on ER-specific chaperones that facilitate proper folding, modifications, and release from the ER (Ulrich et al., 2011; Gidalevitz et al., 2013). For example, GRP (glucose-regulated protein) 78 interacts with the unfolded nascent proteins, contributing to the translocation into the ER (Kleizen and Braakman, 2004). The oxidoreductase enzymes such as PDI, ERp57 or ERp44, catalyze the formation of disulfide bonds between cysteine residues during the folding of many proteins, providing structural stability and promoting the assembly of multi-protein complexes (Bulleid, 2012; Oka and Bulleid, 2013). If the folding capacity of the ER is reduced, proteins tend to accumulate producing a situation called ER stress. Under this challenge, cells up-regulate the expression of chaperones as part of a more complex compartment-specific stress response called the UPR. The UPR is initiated by three ER-resident membrane proteins: IRE1α, PERK, and ATF6α. Briefly, UPR activation results in i) the transcriptional up-regulation of genes coding for chaperones; ii) the attenuation of protein translation; and iii) the increase in the proteasomal and/or autophagy degradation of unfolded/misfolded proteins, through the ERAD (Fujita et al., 2007; Frakes and Dillin, 2017). Due to spatial separation between substrates and degradation systems, ERAD requires retrograde transport through the translocon of unfolded/misfolded proteins from the ER back to the cytosol (Meusser et al., 2005). In this case, substrates are targeted by specific ubiquitin ligases (E3 enzymes) such as the complex HRD1/HRD3 (Hampton et al., 1996; Kaneko and Nomura, 2003). In the end, this coordinate response has two major outcomes: proteostasis restoration or apoptotic cellular death (Gavilán et al., 2009b).

### The Autophagy-Lysosomal System

**FIGURE 2:**
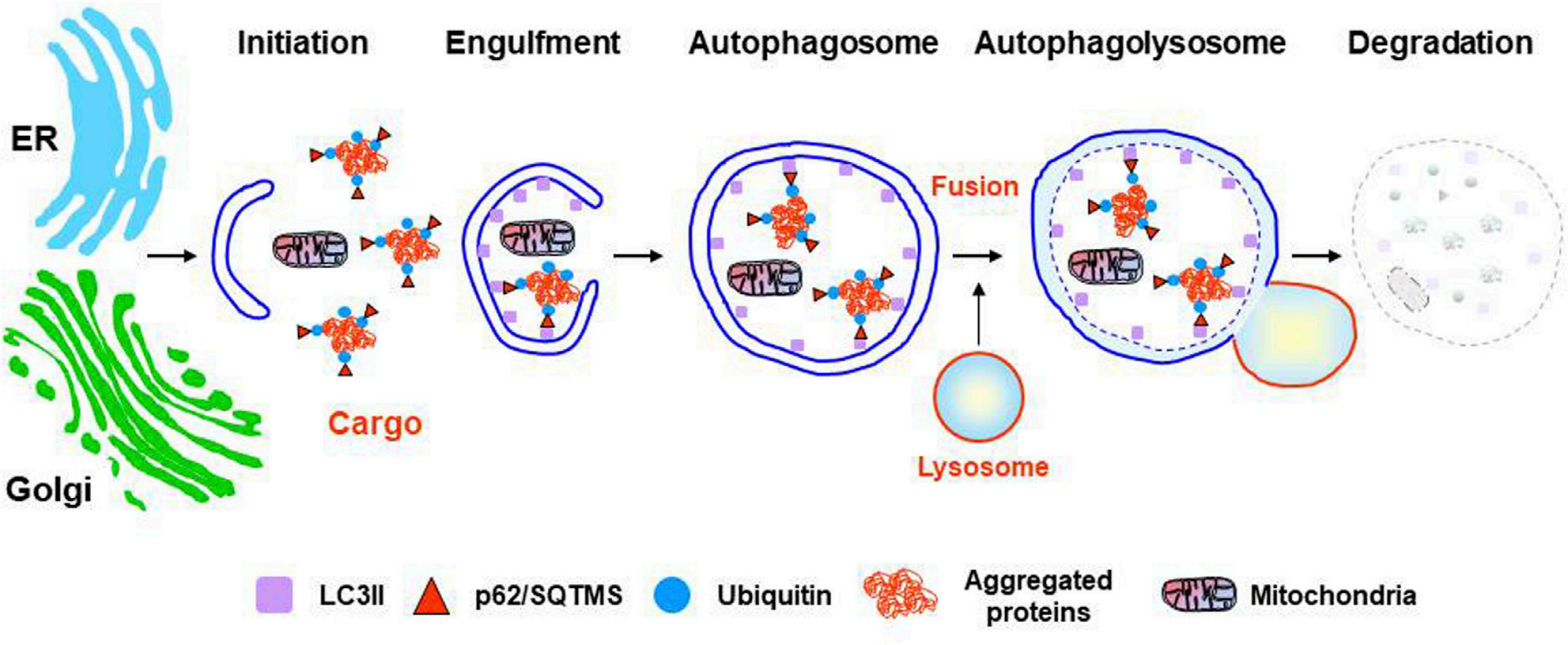
Stages of the autophagy lysosomal pathway. From initiation to resolution the most relevant markers are shown. Two potential sources of autophagosome membranes, but not the only ones, are indicated: ER (endoplasmic reticulum) or Golgi.

The ALS includes three different types of autophagic degradation, all of them ultimately depending on functional lysosomes, but each one acting through different molecular mechanisms (see Galluzzi et al., 2017 for an exhaustive review). Briefly, microautophagy is characterized by the direct capture of cytoplasmic fractions that are taken up by lysosomal membrane invaginations for their degradation (Mizushima et al., 2008). The mechanisms regulating microautophagy in mammalian cells are still poorly known (Mijaljica et al., 2011). CMA is involved in the selective degradation of specific soluble proteins by the lysosomes (Kaushik and Cuervo 2008). It involves neither vesicle formation nor membrane invaginations and participates in the degradation of cytosolic proteins containing the *KFERQ* sequence motif. The CMA-targeting motif is recognized in the cytosol by a chaperone complex including HSc70, HIP, HOP, BAG1, and HSP40, which assists protein translocation into the lysosomal lumen for their degradation in a LAMP2-dependent manner (Cuervo et al., 2014). Finally, macroautophagy (referred to here as autophagy) represents the most relevant form of autophagy (He and Klionsky 2009; Mizushima et al., 2008). Autophagy plays a protective role in various types of stressful contexts such as starvation, protein aggregation, and renewal of damaged or obsolete organelles. It involves the autophagosome formation, a double-membrane vesicle originated by elongation of a *de novo* formed membrane; the sequestration of cargo inside the autophagosome, such as cellular organelles, long-lived proteins and/or aggregated proteins; the seals of the autophagosome; and the transport by the microtubule system, to finally fuse with late lysosomes or endosomes, for cargo degradation, forming autolysosomes. (Ding and Yin, 2008) (Figure 2). The sources of autophagosome membrane in mammal cells are still under debate, and different cellular organelles such as ER, Golgi, mitochondria, or cell membrane, have been found to contribute as membrane donor for autophagosome formation depending on autophagy induction condition (see Wei et al., 2018 for more detailed information). In the last years, different selective forms of autophagic degradation have emerged such as mitophagy, pexophagy, nucleophagy, reticulophagy, ribophagy, aggrephagy, lipophagy, proteaphagy or lysophagy (Galluzzi et al., 2017). Regulation of autophagy is complex, and it has been extensively investigated. The initiation, nucleation, and elongation phases are specifically regulated by different proteins, cellular pathways, and ATGs (see Carlsson and Simonsen, 2015 for a detailed review).

## Functional Cooperation Between the protein Quality Control Systems

**FIGURE 3:**
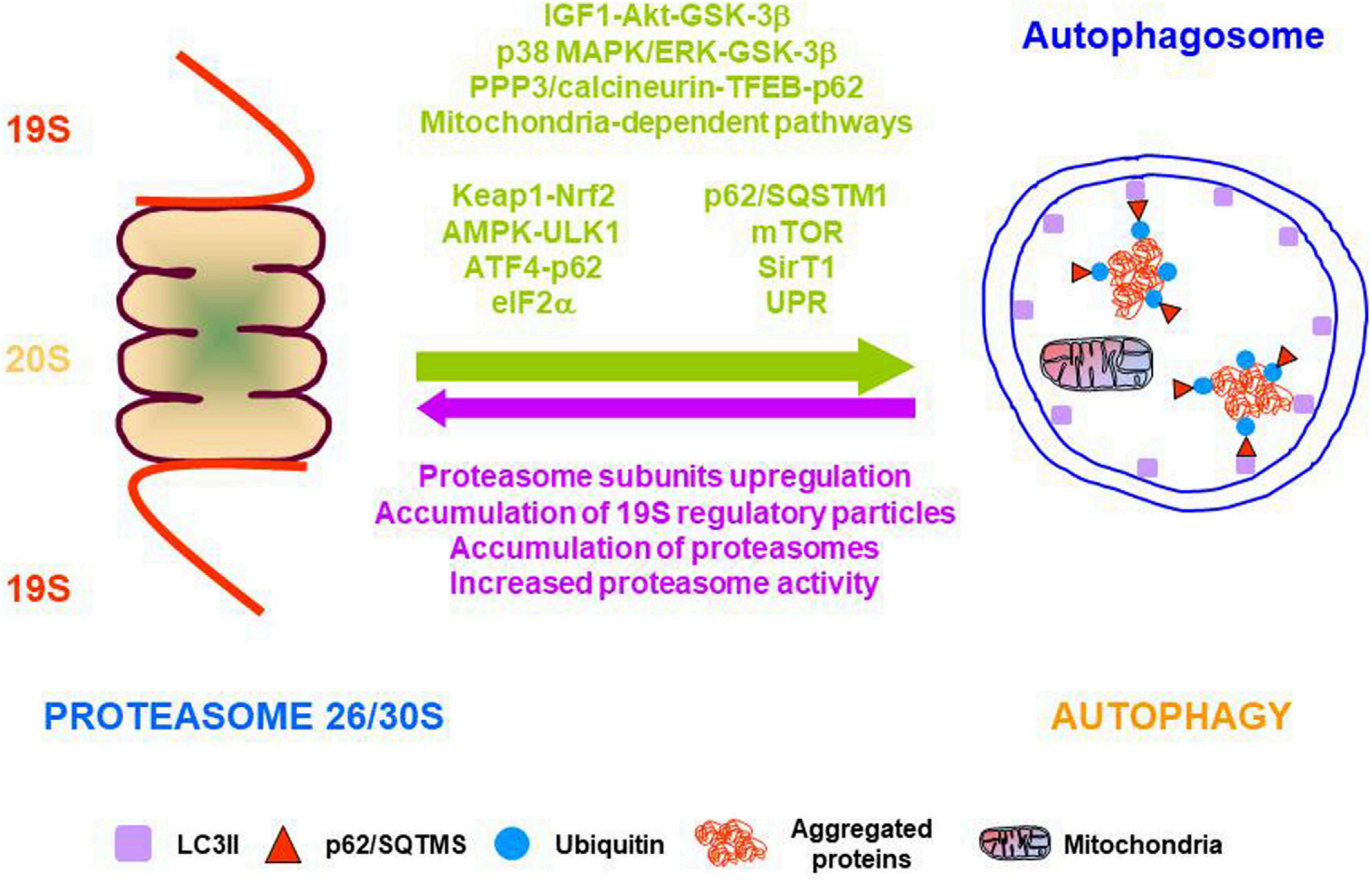
Molecular pathways, proteins, and mechanisms involved in the functional cooperation between the two major proteolytic systems. Arrows indicate the functional cooperation from proteasome to autophagy (green) and from autophagy to proteasome (purple).

Despite molecular mechanisms underlying the interplay between the UPS and autophagy are incompletely understood, there is solid evidence showing functional cooperation between the two major proteolytic systems under stress situations that are mediated by different cellular pathways (Korolchuk et al., 2010). This functional crosstalk allows the integration of many signals to provide a tailored cellular response to each specific cellular challenge (Figure 3). In addition to the functional cooperation of proteolytic systems, evidence also indicates a functional regulation between essential players of cellular proteostasis that cooperate with the two proteolytic systems such as chaperones, UPR, and ERAD.

### Autophagy Compensation for Proteasome Dysfunction

A general overview of available data indicates that proteasome impairment gives rise to autophagy activation. This functional cooperation has been demonstrated both *in vitro* and *in vivo* by different experimental approaches, such as pharmacological or genetic inhibition of the proteasome. However, the cellular pathways involved in this functional crosstalk are very complex and not well understood. A general overview of the pharmacological and genetic interventions is detailed.

#### Pharmacological Approaches

Different compounds are available to block proteasome activity such as MG132, lactacystin, bortezomib, or epoxomicin. Pharmacological inhibition of proteasome with the irreversible proteasome inhibitor lactacystin in rat hippocampus, up-regulated the expression of several ATG, the SQSTM1/p62 (referred to here as p62), and increased autophagic activity through activation of the IGF1-AKT-GSK-3β pathway (Gavilán et al., 2015). A similar effect was described in mouse brain, heart, kidney, and liver using the reversible proteasome inhibitor MG132 or bortezomib, a selective and potent inhibitor of the proteasome with broad anti-tumor activities in many malignancies (Zheng et al., 2011). Other works have shown that MG132 induced autophagy activation in several cell lines through different cell signaling pathways. For example, in MDA-MB-231 human breast epithelial cells, by activating the p38 MAPK/ERK-GSK-3β pathway (Choi et al., 2012), in human breast cancer epithelial cells MCF7, through activation of the AKT-GSK-3β pathway (Gavilán et al., 2013) or in macrophages, epithelial and endothelial cells, by modulating the mitochondrial/AMPK signaling axis (Jiang et al., 2015). Treatment with bortezomib, induced protective autophagy in pancreatic and colorectal cancer cells through AMPK-ULK1 signaling (Min et al., 2014), and in melanoma cells, by both ER and mitochondrial-dependent pathways (Selimovic et al., 2013). Similarly, treatment with MG132 or bortezomib in human colon cancer cells produced ER-stress and UPR dependent autophagy activation. Autophagy was abolished by IRE1α knockdown, or by treatment with the JNK inhibitor SP600125 (Ding et al., 2007), but was independent of XBP-1 signaling (Ding et al., 2007; Rui et al., 2015). Finally, proteasome inhibition with bortezomib in human prostate cancer cells, and immortalized mouse embryonic fibroblasts promoted autophagy activation and upregulated expression of ATG5 and ATG7, which depended on phosphorylation of eIF2α, a downstream element of the PERK arm of the UPR (Zhu et al., 2010).

#### Genetic Approaches

Different targets and strategies have been used to induce proteasomal dysfunction. Elimination of the proteasome activator REGγ in mice produced protective autophagy activation against high-fat diet-induced liver steatosis, mediated by SIRT-1-dependent deacetylation of ATG5 and ATG7 (Dong et al., 2013). Knockdown of the proteasome subunit β2 in mice cardiomyocytes, induced autophagy activation, and increased mTOR expression and ER stress (Kyrychenko et al., 2014). Conditional knock-out mice of the ATPase subunit of the 19S particle Rpt2, increased protective autophagy by activation of the Keap1-Nrf2 pathway in a p62 phosphorylation-dependent manner, in both mouse liver and brain cortical neurons (Kageyama et al., 2014; Ugun-Klusek et al., 2017). However, genetic ablation of *Rpt2* in mouse cardiomyocytes also activated autophagy but through the PPP3/calcineurin-TFEB-p62 pathway (Pan et al., 2020). Knockdown of proteasomal catalytic subunits in human prostate cancer cells and immortalized mouse embryonic fibroblasts promoted autophagy activation and upregulated expression of ATG5 and ATG7 (Zhu et al., 2010). Finally, knockdown of the proteasomal ubiquitin receptors Rpn10 and Rpn13 resulted in autophagy activation and ATF4-p62-dependent clearance of ubiquitinated proteins (Demishtein et al., 2017).

Thus, pharmacological, and genetic data provide solid evidence indicating a robust compensation of autophagy under proteasome dysfunction. Although the mechanisms connecting both proteolytic systems are complex, it is relevant to highlight the role of specific proteins acting as mechanical linkers between both proteolytic systems. They include p62, HDAC6, NBR1, NDP52, OPTN, vcp/p97, Alfy, and BAG proteins (Rogov et al., 2014; Cecarini et al., 2016). For example, the BAG1 and BAG3 proteins participate in protein delivery to the proteasome or the autophagy, respectively (Gamerdinger et al., 2009; Gavilán et al., 2013; Stürner and Behl, 2017). Under normal conditions, most of the polyubiquitinated proteins are degraded by the proteasome in an HSc/HSP70-BAG1 dependent manner (Luders et al., 2000; Demand et al., 2001). But under proteotoxic stress, proteins can accumulate and aggregate leading to increased autophagic activity (Stürner and Behl, 2017). In this case, BAG3, acting in concert with the multi-adapter protein p62, facilitates autophagic degradation (Behl 2016). The p62 protein can bind simultaneously to ubiquitinated proteins, through its UBA domain, to LC3-II, by its LIR domain (Katsuragi et al., 2015) and, to the co-chaperones HSc/HSP70. Thus, p62 would play a pivotal role in the molecular crosstalk between both proteolytic systems, integrating the signals coming from different cellular pathways (Myeku and Figueiredo-Pereira, 2011; Liu et al., 2016; Danieli and Martens, 2018; Aragonès et al., 2020).

### Proteasome Compensation for Autophagy Dysfunction

There is also evidence indicating that autophagy and CMA dysfunction leads to proteasome compensation. For example, in cultured human colon cancer cells, autophagy disruption by RNA interference of ATG genes up-regulated transcriptional expression of proteasomal subunits, including the catalytic β5 subunit, as well as proteasomal activities (Wang et al., 2013). Similarly, in the liver from mice with defective CMA, generated by genetic ablation of LAMP-2A, basal proteostasis was compensated by proteasomal activity due to increased content of the 19S regulatory particle (Schneider et al., 2015). However, there are also data indicating the lack of proteasome compensation for autophagy dysfunction. For example, in fibroblasts from autophagy-deficient mice (*Atg5* ^−/−^), it was not observed any modifications in the three proteasomal activities (Kaushik et al., 2008), as well as in neurons from mice lacking *Atg7* (Komatsu et al., 2006), or in the liver from conditional knockout mice of *Atg7*, where neither proteasomal proteins nor proteasomal trypsin-like activity was modified (Komatsu et al., 2005). Moreover, autophagy inhibition increased proteasome substrates, due to the accumulation of the adaptor protein p62, which inhibited the delivery of ubiquitinated proteins to the proteasome (Korolchuk et al., 2009). Thus, proteasomal compensation from autophagy dysfunction might be organ-dependent.

### Compensation Between Chaperone-Mediated Autophagy and Autophagy

Compensation between the different types of autophagy has also been observed. For example, the decline of CMA by reduction of LAMP-2A expression resulted in the activation of autophagy in cultured mouse fibroblasts (Massey et al., 2006), PC12 cells (Vogiatzi et al., 2008), and mice liver (Schneider et al., 2015). However, in other works CMA down-regulation, produced an accumulation of autophagic vacuoles in HeLa cells (González-Polo et al., 2005), Similarly, dysfunction of CMA in LAMP-2 deficient mice was accompanied by an accumulation of autophagic vacuoles in many tissues, and the impairment of autophagic degradation of long-lived proteins in hepatocytes, suggesting autophagy dysfunction, instead of autophagy compensation (Tanaka et al., 2000). Moreover, a reduction in the proteolytic capacity of lysosomes from LAMP-2 deficient hepatocytes produced autophagy dysfunction, suggesting that LAMP-2 would be somehow necessary for a proper autophagy activity (Eskelinen et al., 2002). Finally, genetic, and pharmacological CMA blockage was not compensated by autophagy activity in neurons (Bourdenx et al., 2021), in mouse embryonic fibroblasts (Eskelinen et al., 2004), or 661W cells (Rodríguez-Muela et al., 2013).

On the contrary, in fibroblasts from autophagy-deficient mice (*Atg5* ^−/−^) (Kaushik et al., 2008), as well as in retinal cells subjected to autophagy inhibition both *in vivo* and *in vitro* CMA was activated (Rodríguez-Muela et al., 2013).

### Chaperones, Unfolded Protein Response, and Endoplasmic Reticulum-Associated Degradation Crosstalk

Functional cooperation between other members of the protein quality control systems, in addition to proteolytic systems, has been also shown.

As stated before, chaperones are associated with protein folding but also participate in protein degradation by both the proteasome and autophagy. Different processes such as the previously mentioned CMA, or chaperone-assisted selective autophagy of aggregated proteins represent two examples of functional cooperation between chaperones and autophagy (Kaushik and Cuervo, 2012). Similarly, chaperones also deliver misfolded proteins for degradation by the UPS, a mechanism called chaperone-assisted proteasomal degradation (Esser et al., 2004; Kettern et al., 2010), where the role of the co-chaperone and ubiquitin ligase CHIP in sorting proteins to refolding or to proteasomal degradation is of central importance (McDonough and Patterson, 2003). As mentioned above, chaperones such as BAG1 and BAG3 participate in protein delivery to the proteasome or the autophagy, respectively, placing chaperones in the middle of the crosstalk between the two cellular protein degradation systems (Park and Cuervo, 2013). However, cooperation of chaperones with proteasomal degradation occurs with cytoplasmic but not with nuclear proteasomal degradation (Samant et al., 2018).

Chaperones have also been shown to regulate the UPR activity in different manners. It is well established that the most abundant ER chaperone GRP78 inhibits UPR activation by binding to the three sensor proteins (Hetz and Papa., 2018). Moreover, the activity of IRE1α is regulated through the binding to IRE1α of several chaperones such as GRP78, PDIA6, and ERdj4, or HSP47, promoting repression or activation of this sensor protein, respectively (Eletto et al., 2014; Amin-Wetzel et al., 2017; Sepulveda et al., 2018). Also, PDIA5 selectively regulates ATF6α activation (Higa et al., 2014), and Erp57, by controlling the oxidative state of PDI, has been found to regulate PERK activity (Kranz et al., 2017). Thus, different cellular chaperones, working independently or together, can specifically regulate the three sensor proteins involved in UPR activation. Reciprocally, UPR activation in response to different stimuli, such as proteasome inhibition, gives rise to transcriptional upregulation of several ER-chaperones as well as ERAD markers to rescue or degrade misfolded proteins, respectively (Paz Gavilán et al., 2006; Walter and Ron, 2011; Sun et al., 2015).

Finally, ERAD might also regulate UPR. In this sense proteasomal degradation of IRE1α is promoted by SEL1L-HRD1 ERAD components, indicating that IRE1α is an ERAD substrate. Depletion of SEL1L or HRD1 in several cell types, increased the amount of IRE1α protein, without affecting transcriptional induction. Importantly, the interaction between IRE1α and SEL1L in the basal condition is dependent on chaperones GRP78 and OS9 (Sun et al., 2015). On the contrary, IRE1α-XBP1 transcriptionally upregulates the expression of SEL1L and HRD1, indicating a bidirectional control between ERAD and UPR.

Altogether, these data indicate that protein quality control systems are functionally interconnected to re-establish proteostasis under proteotoxic stress. Moreover, the molecular versatility observed between the different protein quality control systems, as well as in the different cellular pathways mediating their functional cooperation, suggests that functional cooperation seems to be a cell type-specific process.

## Age-Related Alterations in the Protein Quality Control Systems

The progressive decline in the buffering capacity of the proteostasis network represents one of the molecular hallmarks of aging (López-Otin et al., 2013). However, the biological reasons why the proteostasis network deteriorates during aging are complex and not well understood. A progressive decrease in the activity and efficacy of the protein quality control systems, as well as in the mechanisms mediating the functional cooperation between them, could be the cause of these dysfunctions.

### Molecular Chaperones

A general fact of molecular chaperones during aging is a progressive decline in their amount and/or activity, leading to a lower capacity to cope with cellular stress. A growing body of evidence has demonstrated that many cytosolic and ER chaperones, such as HSP70, HSc70, GRP78, PDI, calnexin, calreticulin, ERp55, ERp57, ERp72, Ero1-like protein alpha, and the family of the ATP-dependent cytosolic chaperones, down-regulate their expression in different cells and tissues, as well as in different organisms including humans (Table 1). However, for some other molecular chaperones, the basal transcriptional expression remains stable in aged cells (Erickson et al., 2006; Paz Gavilán et al., 2006; Brehme et al., 2014; Crum et al., 2015), or even increases (Lee at al., 1999; Brehme et al., 2014; Crum et al., 2015).

**TABLE 1:** Representative molecular chaperones affected by aging in different tissues and organisms.

**HSP70**	**Rat**	
		Mononuclear cells	Deguchi et al. (1988)
		Fibroblasts	Fargnoli et al. (1990)
		Hepatocytes	Heydari et al. (1994)
		Heart	Nitta et al. (1994)
	**Human**	
		Mononuclear cells	Singh et al. (2006)
**GRP78**	**Rat**	
		Hippocampus	Paz Gavilan et al. (2006), Gavilán et al. (2009b)
		Nigral neurons	Salganik et al. (2015)
	**Mouse**	
		Cerebellum	Hussain and Ramaiah, (2007)
		Cortex	
		Kidney	
		Spleen	
		Heart	
		Lung	
		Liver	Rabek et al. (2003), Erickson et al. (2006), Nuss et al. (2008)
**PDI**	**Rat**	
		Hippocampus	Paz Gavilan et al. (2006), Gavilán et al. (2009b)
	**Mouse**	
		Cortex	Naidoo et al. (2008)
		Liver	Rabek et al. (2003), Nuss et al. (2008)
	**Monkey**	
		Chondrocytes	Tan et al. (2020)
**Calnexin**	**Rat**	
		Hippocampus	Paz Gavilan et al. (2006)
	**Mouse**	
		Liver	Erickson et al. (2006)
	**Monkey**	
		Chondrocytes	Tan et al. (2020)
**Calreticulin**	**Mouse**	
		Liver	Rabek et al. (2003)
**ERp55**	**Mouse**	
**ERp57**		Liver	Erickson et al. (2006)
**ERp72**			
**Ero1α**	**Monkey**	
		Chondrocytes	Tan et al. (2020)
**HSc70**	**Rat**	
		Olfactory bulb	Crum et al. (2015)
**ATP-dependent cytosolic chaperones**	**Human**	
		Brain	Brehme et al. (2014)

The decline in the expression of molecular chaperones during aging might reduce the protein folding capacity, increasing the number of unfolded/misfolded proteins in aged cells. For example, the accumulation of ubiquitinated proteins in the aged rat hippocampus following proteasome inhibition was higher in those animals that displayed the lower GRP78 up-regulation (Paz Gavilán et al., 2006). Similarly, in aged human postmortem samples, GRP78 co-localized more frequently with the enzyme tyrosine hydroxylase (healthy dopaminergic neurons), but not with α-synuclein positive neurons (neurodegenerating neurons). By contrast, α-synuclein positive neurons co-localized more frequently with caspase12 (Alladi et al., 2010). Also, inhibition of HSP70 in primary olfactory bulb cultures increased proteotoxicity induced by proteasome inhibition (Crum et al., 2015). However, in lymphoblasts from human centenarians up-regulation of HSP70 in response to heat shock was similar to that observed in young donors, and higher than in aged (non-centenarians) donors (Ambra et al., 2004). These data suggest a correlation of the level of expression of the molecular chaperones with the life span of differentiated cells.

On the other hand, the age-dependent decrease in the content of ER chaperones might affect specifically ERAD activity. In this sense, basal expression of the protein vcp/97, a component of ERAD that participates in the ATP-dependent extraction of misfolded proteins from ER for cytosolic proteasomal degradation, is increased in aged rats, suggesting augmentation of basal ER-stress (Pintado et al., 2017). In summary, considering the many cellular functions in which chaperones are involved, an adequate level of cellular chaperones is of crucial importance to get cellular healthy aging, in order to limit the decrease in tissue and cellular function.

The reasons leading to chaperome down-regulation are currently unknown. In this sense, age-related alterations in general transcriptional expression and translational efficiency have been described in mice (Lee et al., 2000), rats (Wood et al., 2013), and humans (Lu et al., 2004). Thus, future studies focused on age-related modifications in epigenomic mechanisms, such as transcription factor binding, histone marks, heterochromatin formation, and DNA methylation could shed light on the age-related modifications in the mechanisms regulating gene expression (Booth and Brunet, 2016). Moreover, another possibility to explore would be if aggregated proteins in aged cells might catch molecular chaperones, a situation that might contribute to the collapse of proteostasis in aged cells (Yu et al., 2014).

### The Ubiquitin Proteasome System

It is well documented that proteasome activity decreases during normal aging leading to oxidized and/or poly-ubiquitinated protein accumulation. This fact has been described by many groups in many tissues such as the spinal cord, cerebral cortex, kidney, lung (Keller et al., 2000a), hippocampus (Keller et al., 2000a; Gavilán et al., 2009b) liver (Conconi et al., 1996; Keller et al., 2000a) heart (Keller et al., 2000a; Bulteau et al., 2002) epidermis (Bulteau et al., 2000; Petropoulos et al., 2000), lymphocytes (Carrard et al., 2003), muscle (Bardag-Gorce et al., 1999; Radák et al., 2002; Husom et al., 2004; Ferrington et al., 2005), Achilles tendon (Radák et al., 2002), and fibroblasts (Merker et al., 2000), indicating that the gradual decline in the proteasomal activity is a hallmark of aging. However, it should be also noted that Giannini et al., 2013, demonstrated that purified 26S proteasomes from aged rat brain and cerebellum, displayed lower activity than proteasomes from young animals, when using fluorogenic peptides, but exhibited no changes, or even slightly increased activity when a more physiological substrate was used (poly-Ub-model substrate).

The exact mechanisms accounting for the age-dependent decrease in the proteasome activity remain still elusive. For example, structural alterations of the proteasome, as well as reduced expression of proteasome subunits have been described (Lee et al., 1999; Bulteau et al., 2002; Chondrogianni et al., 2003; Gavilán et al., 2009a; Baraibar et al., 2012). On the other hand, the age-related increase in reactive oxygen species, mostly due to mitochondrial dysfunction and dysregulation of anti-oxidant repair mechanisms (Squier, 2001; Rottenberg and Hoke, 2017; Scialo et al., 2017), can also affect proteasome activity by oxidative damage. Every single alpha and beta subunits, as well as regulatory 19S subunits, can be modified by oxidation (Korovila et al., 2017; Lefaki et al., 2017). Irreversible oxidative modifications such as the formation of 4-hydroxynonenal-protein adducts in specific proteasome subunits (Keller et al., 2000b; Petropoulos et al., 2000; Bulteau et al., 2001; Ferrington and Kapphahn, 2004; Wang et al., 2010), or the formation of protein carbonyls in the regulatory subunit S6 ATPase (Rpt5) of the 26S proteasome (Ishii et al., 2005), reduced proteasomal activity. Similarly, the reversible oxidative modification S-glutathionylation has been found in the Rpn2 regulatory subunit of the 26S proteasome, leading to reduced proteasomal degradation of substrates (Zmijewski et al., 2009). But S-glutathionylation of the 20S proteasome has also been proposed to act as a regulatory mechanism to remove oxidized proteins under oxidative stress, by inducing gate opening and enhancing proteasomal activity (Silva et al., 2012). In addition to these oxidative-induced modifications, oxidative stress can also promote other proteasomal modifications such as poly ADP-ribosylation, S-nitrosylation, phosphorylation, or ubiquitination, all of them decreasing proteasomal activity (Kors et al., 2019).

Another relevant role of oxidative stress on proteasome structure and function is the differential susceptibility to oxidative stress displayed by the 20S and the 26S proteasomes. For example, exposure of human hematopoietic K562 cells, yeast, or bovine lens epithelial cells, to several oxidants affected differently the proteolytic activity of the 20S and the 26S proteasomes. Whereas degradation of oxidized proteins by the 20S proteasome was not affected, or even increased, degradation of ubiquitinated proteins by the 26S proteasome was severely reduced or inhibited, suggesting that the 20S proteasome is much more resistant to oxidative stress (Shang and Taylor, 1995; Reinheckel et al., 1998, Reinheckel et al., 2000; Wang et al., 2010).

However, other work in mouse embryonic fibroblasts indicated that under oxidative stress, the 26S proteasome can degrade both oxidized and ubiquitinated proteins, and seemed to be equally resistant to oxidative stress than the 20S proteasome (Haratake et al., 2016). Also, oxidative stress promotes the dissociation of the proteasome from the 26S holoenzymes to free 20S proteasome and the regulatory particle 19S, increasing the 20S/26S ratio (Wang et al., 2010; Grune et al., 2011; Livnat-Levanon et al., 2014; Haratake et al., 2016; Wang et al., 2017). This process is conserved from yeast to human and is mediated, at least in part, by the protein Ecm29 (extracellular mutants 29) (Wang et al., 2010; Haratake et al., 2016; Wang et al., 2017). Considering that oxidatively damaged proteins are mostly degraded by the 20S proteasome, the oxidative-induced increase in the 20S/26S ratio might represent a cellular adaptation to acute oxidative stress (Davies 2001; Grune et al., 2003; Pickering et al., 2010). However, under chronic oxidative stress, as occurring during normal aging, sustained dissociation of the 26S proteasome might favor the accumulation of ubiquitinated proteins due to both reductions of the 26S proteasome, and reduced activity of the oxidized 20S proteasome (Ferrington et al., 2005). In this line, transcriptional up-regulation of several constitutive proteasome subunits has been found in aged rat muscle cells, in parallel with a reduction in the content of the proteasome activating proteins, PA28 and 19S (Ferrington et al., 2005). Also, the aged rat hippocampus increased the content of proteasome subunits, but decreased proteasomal activity, leading to the accumulation of ubiquitinated proteins (Paz Gavilán et al., 2006; Gavilán et al., 2009b). All these data could be compatible with a reduction in the amount of the 26S proteasome in aged cells induced by chronic oxidative stress and/or chronic inflammation (see below). However, other possibilities cannot be ruled out.

In summary, the mechanisms accounting for the age-dependent decrease in the proteasome activity seem to be heterogeneous and probably cell-type specific. The reduction in the number of cellular proteasomes, together with the oxidative damages of specific proteasome subunits could account for most of the age-dependent proteasomal dysfunctions occurring in aged cells. Moreover, proteasomal dysfunction and decreased chaperones activity have synergistic negative effects on the risk of protein accumulation. In turn, accumulated proteins might be prone to form proteins aggregates that cannot be degraded by the proteasome, but might physically block it, leading to a toxic vicious circle especially for post-mitotic cells such as neurons and muscles cells (Gregori et al., 1997; Bence et al., 2001; Grune et al., 2004; Oddo, 2008; Tseng et al., 2008; Höhn et al., 2011).

### The Autophagy-Lysosomal System

Age-related dysfunctions in both autophagy and CMA have also been extensively documented in several tissues such as the liver, brain, and heart (Cuervo and Dice, 2000; Bergamini et al., 2004; Martinez-Vicente et al., 2005; Taneike et al., 2010). The factors accounting for the age-related autophagy dysfunctions in aged cells are also heterogeneous. For example, a reduced formation of autophagic vacuoles, in addition to the delay of fusion of autophagic vacuoles with lysosomes was observed in aged mouse hepatocytes (Terman, 1995). Similarly, in aged mouse retina autophagosome formation, as well as reduction of the LC3 flux, and p62 accumulation was observed (Rodríguez-Muela et al., 2013).

Transcriptional down-regulation of many autophagy genes has been extensively documented in the aged human brain (Lipinski et al., 2010), muscle from aged Fischer 344 rats (Wohlgemuth et al., 2010), aged mouse retina (Rodríguez-Muela et al., 2013), and the hippocampus from aged Wistar rats (Gavilán et al., 2015). Additionally, reduced expression of proteins such as ATG5, ATG12, and Beclin-1 has also been observed in different old tissues (Rodríguez-Muela et al., 2013; Gavilán et al., 2015; Ott et al., 2016). Moreover, epigenetic factors have also been found to regulate autophagy (Füllgrabe et al., 2014; Lapierre et al., 2015; Baek and Kim 2017). For example, autophagy activation was associated with reduced acetylation of H4K16 (Füllgrabe et al., 2013), or increased H3R17 dimethylation (Shin et al., 2016), whereas H3K9 methylation by the histone methyltransferase G9a repressed the expression of LC3B, p62, and other autophagy-related genes (Artal-Martinez de Narvajas et al., 2013). Importantly, hypermethylation in the promoter regions of both the LC3 and ATG5 genes has been observed in macrophages from aged mice, leading to transcriptional downregulation (Khalil et al., 2016).

Finally, as mentioned before, the three arms of the UPR participates in autophagy activation (Kroemer et al., 2010), and basal activity of both UPR and autophagy decreased in aged rats (Paz Gavilán et al., 2006; Naidoo et al., 2008; Gavilán et al., 2009b; Gavilán et al., 2015). For example, the transcription factor sXBP1, a downstream marker of the IRE1α pathway, enhanced autophagy activity by transcriptional upregulation of Beclin-1 in endothelial cells and macrophages (Margariti et al., 2013; Tian et al., 2015), and levels of sXBP1 are decreased in aged rats (Naidoo et al., 2008; Gavilán et al., 2009b).

On the other hand, CMA activity is also decreased in different aged tissues such as the liver, heart, lung, or kidney (Dice, 1982; Cuervo and Dice, 2000; Kiffin et al., 2007; Schneider et al., 2015). In this case, modifications in the lipids of the lysosomal membrane, as well as in the membrane dynamic, and the amount and stability of the LAMP-2A protein at the lysosomal membrane, might be factors contributing to the age-dependent decline of CMA (Kaushik et al., 2006; Kiffin et al., 2007; Zhang and Cuervo, 2008; Rodriguez-Navarro et al., 2012). However, up-regulation of CMA in aged mouse retina has also been observed (Rodríguez-Muela et al., 2013).

Paradoxically, oxidative stress activates autophagy (Scherz-Shouval and Elazar, 2007; Lee et al., 2012) and CMA (Kiffin et al., 2004), probably as a homeostatic response to different acute stressors. However, during aging and under pathological oxidative stress, where oxidative stress became a chronic situation, autophagy activity is blocked as well as the nuclear translocation of TFEB, leading to mitochondrial fission and cellular death (Gavilán et al., 2015; Santin et al., 2016). One potential explanation for these opposed scenarios might be that sustained activation of autophagy could lead to autophagy exhaustion, eventually producing suppression of autophagy (Ho et al., 2016).

In summary, the age-related malfunction of protein quality control systems favors the accumulation of oxidized and/or poly-ubiquitinated proteins and increases cell vulnerability. This aspect is especially relevant in non-dividing cells such as neurons as it has been recently demonstrated (Bourdenx et al., 2021).

### Functional Cooperation

The protein quality control systems form a functionally interdependent network that cooperates to maintain and restore cellular proteostasis under different stress situations. A relevant issue still not deeply analyzed is to evaluate the effect of aging on the functional cooperation between the protein quality control systems. The available data indicate that proteasomal inhibition was efficiently compensated by autophagy activation and resolution in young rat hippocampus, but not in aged animals, leading to proteostasis restoration in young, but protein aggregation and neurodegeneration in old animals (Gavilán et al., 2009b; Gavilán et al., 2015). Also, autophagy compensation in response to CMA dysfunction, observed in the liver from young mice, was lost in old mice (Schneider et al., 2015). Similarly, proteasome inhibition produced the canonical activation of the UPR as well as ERAD induction in young rats, but partial UPR activation (only the PERK pathway) and not ERAD induction in aged animals (Gavilán et al., 2009b; Pintado et al., 2017). Thus, aging seems to harm the functional cooperation between proteolytic pathways, suggesting that compensation might be effective in acute, but not in chronic stress situations such as aging. Indeed, proteasomal degradation in CMA-impaired cells was similar to control cells, but when blockage of CMA was sustained for more than 4 months, proteasomal degradation decreased, due to a reduction in proteasome activities, and to changes in the subunit composition of the 26S proteasome (Massey et al., 2006). Moreover, 26S proteasome dysfunction in *Rpt2* knock-out mice was compensated by increased autophagy in 3 weeks old mice, but this compensation was lost in animals subjected to long-term 26S proteasome dysfunction (6 weeks old mice), due to impairs of the Keap1-Nrf2 pathway (Ugun-Klusek et al., 2017). By contrast, other work found in the retina of old animals that CMA was upregulated in response to autophagy dysfunction (Rodríguez-Muela et al., 2013), suggesting that age-related deterioration of the functional compensation between the different protein quality control systems might be cell-type specific. Finally, an important question that remains to be answered is to know the molecular mechanisms underlying the age-dependent decline in functional cooperation. Current data indicate that defective signaling of the IGF1-AKT-GSK-3β-β-catenin pathway could account for the decrease in autophagy compensation in rat hippocampus in response to proteasome inhibition (Gavilán et al., 2015). And disrupted signaling of TFEB might be involved in defective compensation of autophagy in response to CMA dysfunction in liver mouse (Schneider et al., 2015).

In summary, these data support the idea that proteolytic systems form an intricate network that compensates each other to restore proteostasis. Age-dependent alterations of this functional crosstalk might compromise cellular viability. Since many different cellular pathways can modulate the functional cooperation between the protein quality control systems, future works using different cell types and stressors will be necessary, to better understand how aging is affecting functional cooperation. Because proteostasis alteration is also characteristic of some age-dependent pathological disorders, the identification of prevalent factors contributing to the disruption of the functional cooperation between proteolytic systems will become a major challenge for biomedicine and geroscience for the coming years.

## Inflammation

Aging is also characterized by the presence of a low-grade chronic inflammation status called inflammaging (Franceschi et al., 2000). For example, the level of the pro-inflammatory cytokines IL-1β, IL-6, TNF-α, and C-reactive protein, are increased in aged organisms (Gavilán et al., 2007; Minciullo et al., 2012; Barrientos et al., 2015; Scheinert et al., 2015). Moreover, activated microglial cells (Gavilán et al., 2007), macrophages infiltration (Wolfe et al., 2018), as well as alterations in T cells and macrophages function (Vaughan and Peters, 1974; Sheng et al., 1998; Conde and Streit, 2006; Norden and Godbout, 2013), are characteristic of aging. Thus, aged cells are exposed to a chronic inflammatory environment that could affect their homeostatic response.

### Inflammation and the Protein Quality Control Systems

A growing body of evidence indicates a complex and bidirectional association between protein quality control systems and inflammation. For example, Th1 or Th2 cytokines stimulated or inhibited autophagy, respectively (Wu et al., 2016). Also, TNF-α modulated proteasome and autophagy function in human skeletal muscle cells (Keller et al., 2011), and in synovial fibroblasts from rheumatoid arthritis (Connor et al., 2012). LPS-induced neuroinflammation produced ER-stress, altered proteasome and autophagy activity, and down-regulated ERAD markers (Liu X.,D. et al., 2012; Pintado et al., 2017). Moreover, up-regulation of ERAD markers induced by proteasome inhibition was abolished by LPS-induced inflammation (Pintado et al., 2017). Also, UPR activation has been found to increase the production of inflammatory cytokines. The three arms of the UPR: IRE1α-TRAF2, PERK-eIF2α, PERK-GSK-3, and ATF6-CREBH can activate the transcription factor NFκ-B, which has a pivotal role in the onset of inflammation (Salminen et al., 2009; Vallabhapurapu and Karin, 2009 ). For example, NF-κB activation and TNF-α synthesis, induced by ER stress, were impaired in IRE1α knockdown mouse embryonic fibroblasts (Hu et al., 2006). Also, activation of Toll-like receptors in macrophages induced specifically the IRE1α-XBP1 pathway and cytokine production (Martinon et al., 2010). However, activation of Toll-like receptors suppressed CHOP expression despite PERK activation (Woo et al., 2012). Moreover, recent work demonstrates that XBP-1 silencing in macrophages inhibited the production of IL-1β, TNF-α, and IL-6 induced by TREM-1 activation, and reciprocally, TREM-1 activation-induced UPR in primary macrophages (Dong et al., 2021).

On the other hand, cytokines such as α-interferon, γ-interferon, or TNFα promote the replacement of the catalytic subunits of the 20S proteasome (β_1_, β_2_, and β_5_), by the inducible subunits β_1i_, β_2i_, and β_5i_ (Gaczynska et al., 1993; Aki et al., 1994; Rivett et al., 2001; Gavilán et al., 2012; Jimenez-Guardeño et al., 2019). These subunits associate with the proteasome activator PA28 complex (also named 11S), forming a proteasome isoform called immunoproteasome (Chondrogianni and Gonos, 2007; Gavilán et al., 2012). The immunoproteasome is constitutively expressed in immune cells and compared with the 20S proteasome has different proteolytic activities. Among other functions, the immunoproteasome participates in antigen presentation (Yang et al., 1995; Strehl et al., 2005; Chapiro et al., 2006), γ-interferon-mediated microglial activation (Moritz et al., 2017), cytokine production by microglial cells (Wagner et al., 2017), or the maintenance, expansion, and regulation of T-cell population (Zaiss et al., 2008; Muchamuel et al., 2009; Moebius et al., 2010).

However, in addition to providing peptides for antigen presentation, and other immune functions, the immunoproteasome degrades nascent oxidant damaged proteins, also known as DRiPs (Seifert et al., 2010; Opitz et al., 2011), increases the cellular proportion of hydrophobic peptides (Gaczynska et al., 1996; Cascio et al., 2001; Chapiro et al., 2006 ; Paz Gavilán et al., 2006; Gavilán et al., 2009a), and regulates autophagy (Pintado et al., 2017; Karim et al., 2020). Thus, the immunoproteasome plays an important general role in the maintenance of cellular proteostasis under acute inflammation.

On the contrary, the protein quality control systems can also regulate the inflammatory response. For example, selective inhibition of the immunoproteasome subunit β_5i_ blocked the production of interferon-γ and IL-2 by T cells, and interleukin-23 by activated monocytes (Muchamuel et al., 2009). Moreover, the recently described proteasome-associated autoinflammatory syndromes, such as Nakajo-Nishimura syndrome (Arima et al., 2011), lipodystrophy (Kitamura et al., 2011), or chronic atypical neutrophilic dermatosis (Liu Y. et al., 2012), are caused by inherited and/or *de novo* loss-of-function mutations affecting both constitutive and immunoproteasome subunits (α7, β2, β7, β1i, β2i, β5i) (Sarrabay et al., 2020), or chaperone proteins (POMP, PAC2) (Brehm and Krüger, 2015; Poli et al., 2018; de Jesus et al., 2019). Finally, autophagy disruption is related to increased ER stress and the production of pro-inflammatory molecules (Ghosh et al., 2016).

In summary, these data indicate that inflammation and proteostasis are two processes mutually influenced. This functional relationship might be useful to fine-adjust the activity of both cellular processes to acute stress situations. However, chronic activation of inflammation might negatively affect the protein quality control systems avoiding proteostasis restoration.

### Inflammation and Proteostasis in Aged Cells

Because aging is associated with a low grade of chronic inflammation, the modulation exerted by inflammation on cellular proteostasis might be particularly relevant in aged cells. For example, the immunoproteasome, which is not expressed in cells from young animals, is expressed in rat and human aged cells from several tissues (Ferrington et al., 2005; Mishto et al., 2006; Gavilán et al., 2007; Gavilán et al., 2009a; Wagner et al., 2017). Moreover, proteasome turnover is regulated by neuroinflammation. Whereas in young rats, irreversibly damaged proteasomes were replaced with constitutive proteasomes, in aged rats they were replaced with immunoproteasomes (Gavilán et al., 2012). Also, the content of small hydrophobic peptides, mostly produced by the immunoproteasome (Gaczynska et al., 1996; Cascio et al., 2001; Chapiro et al., 2006 ; Gavilán et al., 2009a), increased in the aged rat hippocampus. And, it has been also shown that inflammation increased the production of DRiPs, which are preferentially degraded by the immunoproteasome (Seifert et al., 2010). All these data indicate that chronic inflammation provides a cellular environment prone to protein aggregation (Pintado et al., 2012; Gavilán et al., 2015; Pintado et al., 2017).

Most of the age-related alterations observed in cellular proteostasis are often reproduced in young animals following LPS injection. For example, LPS induced the expression of the immunoproteasome and decreased proteasomal activity leading to the accumulation of polyubiquitinated proteins in pyramidal neurons (Pintado et al., 2012). Also, LPS increased the content of hydrophobic peptides (Gavilán et al., 2009a), induced autophagic activation, activated the UPR, and decreased the expression of ERAD markers (Pintado et al., 2017). Finally, the combination of inflammation and proteasome inhibition in young rat hippocampus reduced the UPR activation and the expression of ERAD markers (Pintado et al., 2017) and produced a similar degree of neurodegeneration to that observed in aged animals subjected only to proteasome inhibition (Gavilán et al., 2009b; Pintado et al., 2012).

All these data indicate that inflammation and proteostasis alteration should be considered as synergistic negative factors that might increase cell vulnerability in aging. This is especially relevant in the context of some age-related pathologies such as obesity, hypertension, diabetes, and neurodegenerative disorders, all of them characterized by oxidative stress and inflammation. However, having in mind the complexity in the reciprocal influences between inflammation and the different protein quality control systems, as well as the cell specificity of these interactions, further studies in the context of aging will be necessary to better understand the synergistic negative effects of these two processes.

## Conclusion

**FIGURE 4:**
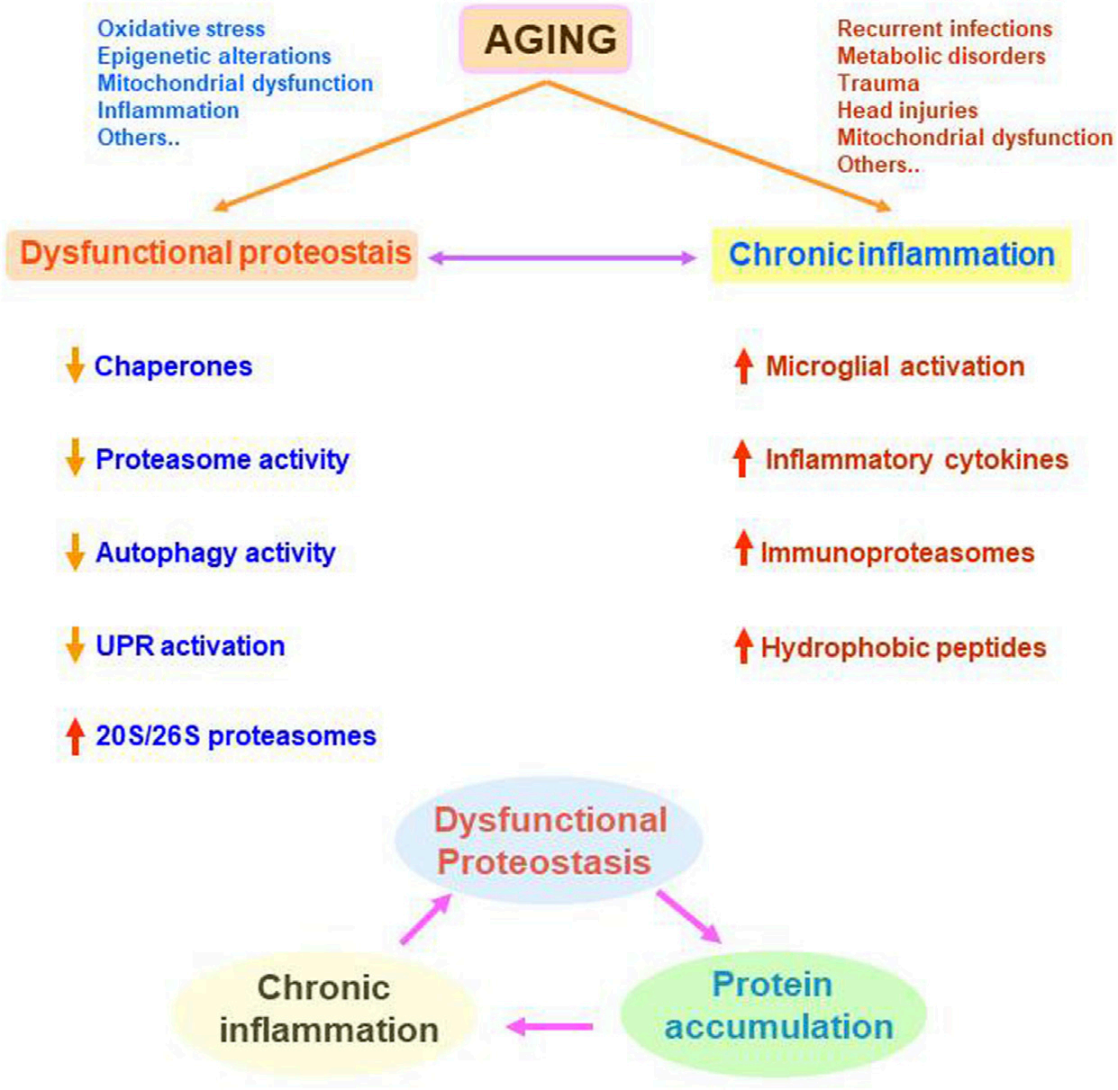
Representation of the age-related alterations in cellular proteostasis and inflammation and their potential synergistic negative effects. Many different situations occurring all along the life can be involved in dysfunctional proteostasis and/or chronic inflammation. These two processes can be modulating each other leading to a vicious circle. Dysfunctional proteostasis leads to protein accumulation that can potentiate or sustains chronic inflammation. In turn, chronic inflammation potentiates dysfunctional proteostasis.

The progressive and irreversible disruption of physiological functions, as a consequence of age-dependent systemic dysregulation, produces aging cell and whole-organism deterioration. Aging is a multifactorial process and here, I have reviewed how cellular proteostasis and inflammation become altered during aging (Figure 4). For example, chronic systemic diseases, recurrent infections, or metabolic disorders, might be factors promoting chronic inflammation throughout life, which in turn represent a hallmark of neurodegenerative disorders such as Alzheimer’s and Parkinson’s diseases. Inflammation and proteostasis regulate each other. This reciprocal regulation might be useful during acute stress situations to control the homeostatic response. However, during chronic inflammation and/or chronic proteostasis alteration, these two stressful situations might be reciprocally potentiated, increasing cell vulnerability.

Even though aging is a progressive and irreversible process, modulation of inflammation and oxidative stress might result in a slowdown of the cellular proteostasis affectation. Therefore, a healthy lifestyle is pivotal to reach successful aging. Promising preventive strategies such as healthy nutrition, and mainly, regular physical activity should be incorporated into our daily lifestyle, to prevent most of the age-related pathologies. For example, caloric restriction, without malnutrition, is the most powerful non-genetic intervention for extending longevity and healthspan in multiple animal models (Fontana et al., 2010). It has been found that caloric restriction reduced cellular senescence and mitochondrial dysfunction, as well as activated autophagy and promoted DNA repair (Fontana et al., 2018). Also, caloric restriction reversed the abnormal patterns of cell communication, and the excessive proinflammatory ligand-receptor interplay, observed during aging (Ma et al., 2020). In addition to caloric restriction, another promising dietary strategy for reducing oxidative damage and inflammation is intermittent fasting (Longo and Mattson, 2014).

On the other hand, the regular practice of a physical activity is one of the most promising anti-aging strategies (Rebelo-Marques, et al., 2018). It is widely accepted that physical activity has positive effects on the aging immune system. Physical activity has anti-inflammatory properties (Gleeson et al., 2011), ameliorates metabolic health in older people (Pedersen, 2006), reduces inflammaging and immunosenescence (Weyh et al., 2020), and induces autophagy by modulating the IGF-1/AKT/mTOR, and AKT/FOXO3A signaling pathways (Kim Y.,A. et al., 2013; Luo et al., 2013), among other benefits. Moreover, epidemiological studies have found that physical inactivity is associated with systemic low-grade inflammation (Parsons et al., 2017).

Although aging is an irreversible process, it can be modulated. Strategies combining diet and physical activity will allow us to reduce, at the molecular level, the most harmful effects of aging leading to disability and frailty.

## Abbreviations

ALFY, Autophagy‐linked FYVE protein; ALS, Autophagy‐lysosomal system; AMPK, AMP activated protein kinase; ATF4, Activating transcription factor 4; ATF6α, Activating transcription factor 6α; ATG, Autophagy‐related genes; BAG, Bcl-2-associated ethnogeny; CHIP, Carboxyl terminus of HSc70-interacting protein; CHOP, C/EBP‐homologous protein; CMA, Chaperone-mediated autophagy; CREBH, Cyclic AMP Response Element-binding Protein H; DRiPs, Defective ribosomal products; eIF2α, Eukaryotic translation initiation factor 2 alpha; ER, Endoplasmic reticulum; ERAD, Endoplasmic reticulum‐associated degradation; ERK, Extracellular signal‐regulated kinase; ERdj4, Endoplasmic reticulum‐localized DnaJ 4; ERp, Endoplasmic reticulum protein; FOXO3A, Forkhead Box O3A; GRP, Glucose-regulated protein; GSK, Glycogen synthase kinase; HDAC6, Histone deacetylase 6; HECT, Homologous to the E6-AP C-terminus; HIP, Hsp70 interacting protein; HOP, Hsp70‐Hsp90 organizing protein; HRD, Hidroxi-metil-glutaril-CoA reductase degradation protein; HSP, Heat shock proteins; HSc, Heat shock cognate; IGF1, Insulin-like growth factor‐1; IL‐1β, Interleukin-1 beta; IRE1α, Inositol-requiring enzyme 1α; JNK, c‐Jun N-terminal kinase; Keap1,Kelch‐like ECH‐associated protein 1; LAMP, Lysosome-associated membrane protein; LC3‐II, Microtubule‐associated protein 1 light chain 3 alpha-II; LIR, LC3-interacting region; LPS, Lipopolysaccharide; MAPK, Mitogen-activated protein kinase; MCF7, Michigan Cancer Foundation-7; MDA-MB, M.D. Anderson-Metastasis breast cancer; mTOR, Mechanistic target of rapamycin; NBR1, Neighbor of BRCA1 gene 1; NDP52, Nuclear dot protein 52; NFκ-B, Nuclear factor kappa-light-chain-enhancer of activated B cells; Nrf2, Nuclear factor erythroid 2-related factor 2; OPTN, Optineurin; PA28, Proteasome activator 28; PAC2, Proteasome Assembly Chaperone 2; PDI, Protein disulfide isomerase; PERK, Protein kinase RNA-like ERK; POMP, Proteasome maturation protein; PPP3, Protein phosphatase 3; Rpn, Regulatory particle non-ATPase; Rpt, Regulatory particle ATPase; RING, Really interesting new gene; SIRT-1, Silent information regulator two ortholog-1; SEL1L, Suppressor/enhancer of Lin-12-like; SQSTM1, Sequestosome 1; TNF-α, Tumor necrosis factor-alpha; TRAF2, TNF receptor-associated factor 2; TREM-1, Triggering receptor expressed on myeloid cells-1; TFEB, Transcription factor EB; ULK1, Unc-51 like autophagy activating kinase 1; UPR, Unfolded protein response; UPS, Ubiquitin proteasome system; VCP, Valosin-containing protein; XBP-1, X-box binding protein 1.
